# Fast-tracked and optimised: The impact of a one-stop lung cancer clinic on patient outcomes

**DOI:** 10.1016/j.fhj.2025.100474

**Published:** 2025-09-26

**Authors:** Jonathan Hiu Nian Chung, Kavita Sivabalah, George Hulston, Patrick Goodley, Lisa Galligan-Dawson, Sarah Lyon, Felice Granato, Vijay Joshi, Aleksander Mani, David Woolf, Kathryn Banfill, Claire Barker, Jennifer King, Cassandra Ng, Helen Huddart, Rebecca Stephens, Siobhan Keegan, Karen Peplow, Julie Watts, Kirsty Rowlinson-Groves, Jack Murphy, Kath Hewitt, Matthew Evison

**Affiliations:** a Lung Cancer & Thoracic Surgery Directorate, Wythenshawe Hospital, Manchester University NHS Foundation Trust, Manchester, UK; b Greater Manchester Cancer Alliance, The Christie NHS Foundation Trust, Manchester, UK; c Clinical Oncology Department, The Christie NHS Foundation Trust, Manchester, UK; d Prehab4Cancer, Salford Council, Greater Manchester, UK; e Manchester Academic Health Science Centre (MAHSC), Faculty of Biology, Medicine & Health, University of Manchester

**Keywords:** Lung cancer, Thoracic surgery, Thoracic oncology, Radiation oncology

## Abstract

**Introduction:**

Patients with resectable early-stage lung cancer who are at higher risk from thoracic surgery often face complex pathways to make a treatment decision, resulting in negative outcomes.

**The solution:**

The Greater Manchester One-stop Lung Cancer Clinic (GM-OSLCC) provides a multidisciplinary service of thoracic surgeons, anaesthetists, clinical oncologists, physicians, specialist nurses alongside prehabilitation, tobacco dependency and frailty specialists to support treatment decisions in a single clinic visit. Outcomes were compared between a pre-implementation cohort (*n* = 94) and a post implementation cohort (*n* = 114).

**Outcomes:**

The median time from referral to decision to treat was reduced from 20 days (interquartile range (IQR): 13–32) to 4 days (IQR: 3–8, *p* ≤ 0.001). The GM-OSLCC increased the uptake of prehabilitation (*p* = 0.036), frailty interventions (*p* < 0.001), tobacco dependency treatment (*p* < 0.001) and malnutrition interventions (*p* < 0.001). The average number of outpatient clinic visits reduced from 2.4 to 1 and 12-month mortality reduced from 12.8% to 3.5% (*p* = 0.013).

**Discussion:**

This one-stop model of care has delivered significant pathway improvements and increased the uptake of optimisation interventions, leading to significantly reduced 12-month mortality.

## Introduction

Lung cancer is the leading cause of cancer-related deaths globally, responsible for nearly 2 million deaths annually.^1^ The gold standard treatment for early-stage lung cancer is surgical resection, provided that the patient has adequate physiological reserve.^2^ However, many patients have chronic cardiorespiratory comorbidities, which increase surgical risks. For those deemed medically inoperable, stereotactic ablative radiotherapy (SABR) offers a viable alternative with acceptable long-term control rates.^3^, ^4^, ^5^ A complex scenario arises when patients with resectable early-stage lung cancer are at higher risk for surgery, but are not inoperable. A previous UK randomised controlled trial (RCT) failed to demonstrate feasibility in randomising such patients between surgery and radiotherapy.^6^ In the absence of clear evidence, these patients must navigate a difficult decision-making process through consultations with both thoracic surgery and oncology teams, which can lead to delays. Delays in the lung cancer pathway, particularly in early-stage surgery, negatively impact outcomes.^7^, ^8^, ^9^, ^10^ Thus, patients with resectable but high-risk early-stage lung cancer face a complex and delayed decision-making process that adversely affects outcomes.

## Methodology

Greater Manchester (GM) is an integrated care system (ICS) in the north-west of England, with GM Cancer as its strategic cancer arm. Delays in the lung cancer pathway, particularly for resectable early-stage lung cancer patients at high surgical risk, have long threatened cancer performance. To address this, GM Cancer established a multidisciplinary taskforce, including patient representatives, to design, implement and evaluate the ‘GM One Stop Lung Cancer Clinic’ (GM-OSLCC).

### Case study setting

GM has a regional thoracic surgery centre for all lung cancer resections, with radiotherapy provided by a separate tertiary cancer centre. Before the new service, patients with resectable lung cancer at higher surgical risk from across GM (seven hospital trusts) underwent dual referrals to both thoracic surgery and oncology or started with thoracic surgery assessment. They would then be listed for discussion at a weekly ‘High-risk MDT’ (HRMDT), followed by additional tests, anaesthetic and oncology consultations, and potentially multiple clinic visits, risking significant delays and poor patient experience.

### Pathway redesign

This pathway redesign had several objectives: 1) to eliminate delays in complex pathways, 2) to provide an exceptional experience of care, and 3) to reduce treatment-related adverse events through comprehensive optimisation interventions.

### The GM One-stop Lung Cancer Clinic

The GM taskforce implemented a ‘one-stop’ clinic model, allowing patients to consult all relevant specialists in a single visit and make treatment decisions on the same day. Key features include: 1) a centralised clinic at the thoracic surgical centre with a thoracic surgeon, anaesthetist, oncologist, onco-geriatrician and respiratory physician, supported by exercise specialists,^11^^,^^12^ tobacco dependency advisers^13^ and cancer nurse specialists, 2) a comprehensive nurse-led assessment on arrival to screen for frailty, cognition, nutrition, alcohol/tobacco use, medical history and preferences, including a video-recorded 30-sec sit-to-stand test, 3) a pre-clinic MDT to review referral information, test results and treatment options, 4) consultations with all necessary clinicians, with a dedicated nurse advocate for each patient, 5) on-the-day consent and planning, with surgery candidates receiving pre-op tests and surgery dates, and radiotherapy patients scheduled for planning scans.

### Referral criteria

Standardised referral criteria were agreed for identified patients at higher risk from thoracic surgery (Box 1 Supplementary materials).

### Evaluation

The GM-OSLCC launched in June 2022 on a once-a-week basis. A pre-implementation cohort (patients discussed at the surgical centre HRMDT from January to May 2022) was compared with a post-implementation cohort (patients referred to GM-OSLCC from July to December 2022). Comparisons of key outcomes were made between pre- and post-implementation cohorts. The statistical strength of association between each variable and cohort were evaluated with statistical tests performed using R version 4.3. Pearson’s χ^2^ test was generally used for categorical variables, with Fisher’s exact test used with low expected frequencies (<5). Continuous variables with symmetric distributions were reported as means (±standard deviation) and strengths of association tested using the Welch two sample t-test. Continuous non-symmetric variables were reported as medians (quartile 1–quartile 3), and comparisons tested with the Wilcoxon rank sum test. A second post-implementation cohort (January–Sept 2023), following expansion to bi-weekly clinics, was also analysed for pathway times only.

## Outcome

The pre- and post-implementation cohorts included 94 and 114 patients respectively. The second post-implementation phase, when the clinic expanded to twice weekly, included 161 patients.

There were statistically significant differences in the pre- and post-implementation cohorts, with patients seen in the GM-OSLCC having worse performance status, lower post-operative predicted diffusion capacity of the lungs for carbon monoxide (DLCO), lower incremental shuttle walk test distances and higher prevalence of moderate-high risk of malnutrition (Table 1).

**Table 1:** Comparison of patient demographics, clinical variables and physiological variables in the pre- and post-implementation cohorts.

	HRMDT, N = 94	One stop, N = 114	*p*-value^a^
Age, mean (±SD)	70 (±8)	71 (±8)	0.19
Sex, *n* (%)			0.28
Female	40 (43)	57 (50)	
Male	54 (57)	57 (50)	
IMD quintile, *n* (%)			0.063
1	43 (46)	46 (41)	
2	13 (14)	30 (27)	
3	9 (9.7)	9 (8.1)	
4	9 (9.7)	15 (14)	
5	19 (20)	11 (9.9)	
(Missing)	1	3	
WHO performance status, *n* (%)			<0.001
0	28 (30)	17 (15)	
1	56 (60)	58 (51)	
2	10 (11)	37 (32)	
3	0 (0)	2 (1.8)	
Lung cancer stage (TNM8), *n* (%)			0.17
I	52 (55)	77 (68)	
II	22 (23)	24 (21)	
III	17 (18)	12 (11)	
IV	1 (1.1)	1 (0.9)	
Metastasis from extrapulmonary cancer	2 (2.1)	0 (0)	
PPO-FEV1 % predicted, mean (±SD)	62 (±16)	61 (±18)	0.56
(Missing)	1	3	
PPO-DLCO % predicted, mean (±SD)	53 (±16)	48 (±14)	0.014
(Missing)	4	5	
ISWT, m, mean (±SD)	366 (±151)	292 (±142)	0.002
(Missing)	21	28	
Echocardiographic abnormality, *n* (%)			0.14
Normal	63 (67)	86 (83)	
Mild	7 (7.4)	3 (2.9)	
Moderate	9 (9.6)	7 (6.8)	
Severe	2 (2.1)	7 (6.8)	
(Missing)	13	11	
CFS, *n* (%)			0.009
1	1 (2.3)	2 (1.8)	
2	12 (27)	11 (9.6)	
3	22 (50)	47 (41)	
4	6 (14)	41 (36)	
5	3 (6.8)	9 (7.9)	
6	0 (0)	4 (3.5)	
(Missing)	50	0	
BMI, kg/m^2^ mean (±SD)	26.8 (±6.2)	26.1 (±5.6)	0.40
(Missing)	13	1	
MUST, *n* (%)			0.029
0	54 (86)	95 (83)	
1	3 (4.8)	0 (0)	
2	6 (9.5)	19 (17)	
(Missing)	31	0	

IMD, index of multiple deprivation; WHO, World Health Organization; PPO-FEV1, predicted post-operative forced expiratory volume in 1 sec; PPO-DLCO, predicted post-operative diffusion capacity of the lungs for carbon monoxide; ISWT, incremental shuttle walk test; CFS, clinical frailty scale; BMI, body mass index.

a Welch two-sample t-test; Pearson’s chi-squared test; Fisher’s exact test; Wilcoxon rank sum test.

**Fig. 1:**
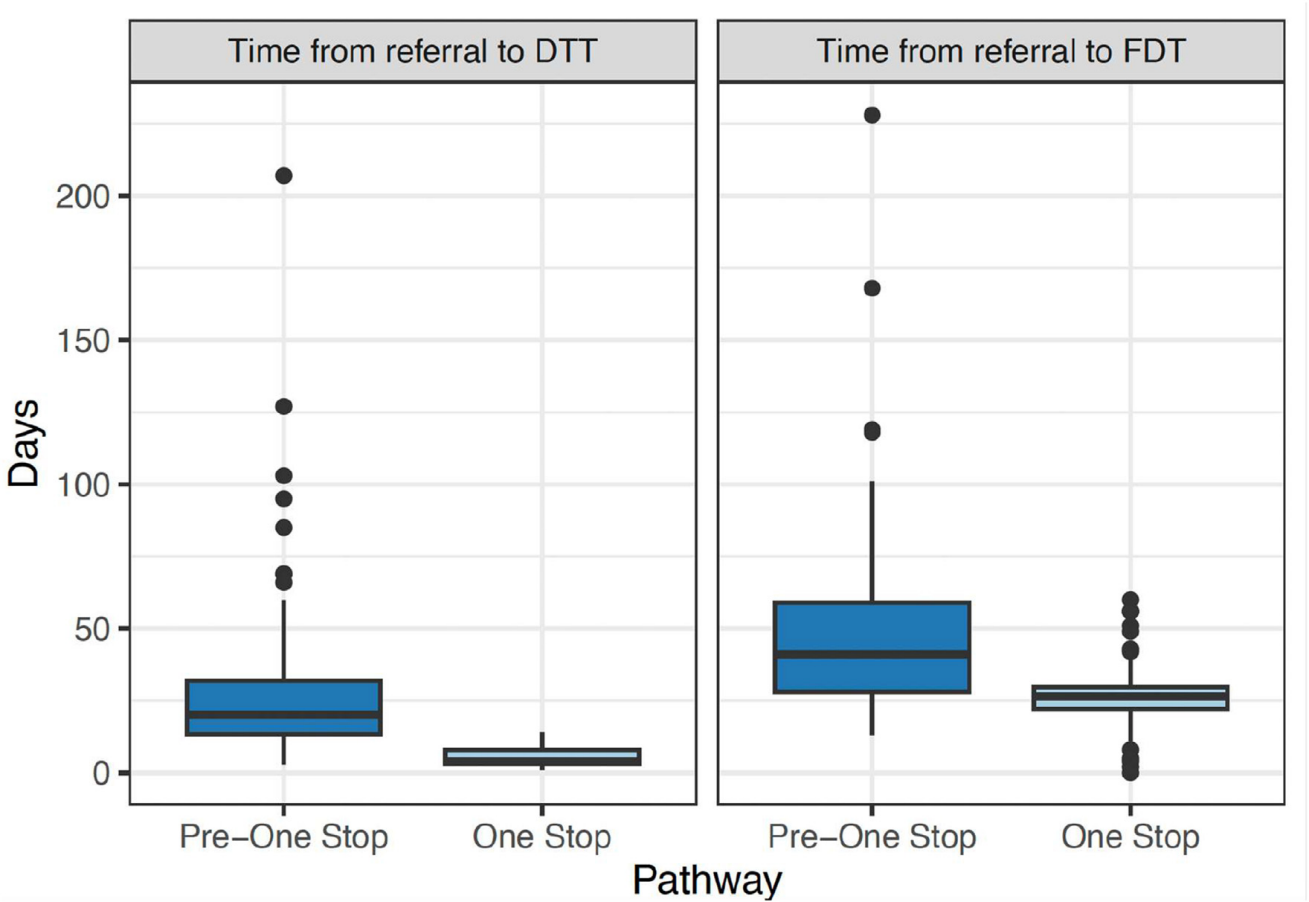
Box plots comparing the time from referral to decision to treat (DTT, left panel) and time from referral to final decision/treatment (FDT, right panel) before and after the introduction of the GM One-stop Lung Cancer Clinic (*p* < 0.001).

The median time from referral to decision to treat (DTT) in the pre-implementation cohort was 20 days (IQR 13–32) vs 4 days (IQR 3–8) in the post-implementation cohort (*p*-value < 0.001, Table 2, Fig. 1). The median time from referral to first definitive treatment (FDT) in the pre-implementation cohort was 41 days (IQR 28–59) vs 26 days (IQR 22–30) in the post-implementation cohort (*p*-value < 0.001, Table 2). The proportion of patients who made a treatment decision on the day of attendance at the GM-OSLCC was 88% (100/114) compared to 0% (0/94) in the pre-implementation cohort. In the second implementation phase, the median time from referral to DTT was 5 days (IQR 4–8) and the proportion of patients who made a treatment decision on the day of attendance at the GM-OSLCC was 85% (137/161).

**Table 2:** Impact on complex pathway delivery and delays of the GM One-stop Lung Cancer Clinic.

	HRMDT, N = 94	One stop, N = 114	*p*-value^a^
Days from referral acceptance to decision to treat (DTT), median (IQR)	20 (13–32)	4 (3–8)	<0.001
Days from referral acceptance to first definitive treatment (FDT), median (IQR)	41 (28–59)	27 (22–30)	<0.001

a Wilcoxon rank sum test.

The GM-OSLCC lead to a significant increase in the uptake of prehabilitation (68%, 64/94 vs 81%, 92/114, *p* = 0.036, Table 3), prehabilitation completion (52%, 49/94 vs 65%, 74/114, *p* = 0.062), frailty screening (48%, 45/94 vs 100%, 114/114), frailty interventions (4%, 4/94 vs 41%, 46/114, *p* < 0.001), tobacco dependency treatment (15%, 5/34 vs 91%, 30/33, *p* < 0.001), nutrition interventions in moderate-high risk patients (11%, 1/9 vs 100%, 19/19, *p* < 0.001) and alcohol dependency screening and compliance with intervention protocol (0%, 0/94 vs 100% 114/114, *p* < 0.001).

**Table 3:** Screening and uptake of interventions for optimising patients in preparation for treatment pre- and post-implementation of the GM One-stop Lung Cancer Clinic.

		Pre-one-stop implementation N = 94	Post-one-stop implementation N = 114	*p*-value
Prehabilitation (prehab4cancer)	Uptake of prehab	64/94 (68%)	92/114 (81%)	0.036
	Completion of prehab	49/94 (52%)	74/114 (65%)	0.062
	Median number of prehab sessions	6 (3–8)	4 (2–7)	0.046
Frailty *n* (%)	Patients screened for frailty (documented CFS)	44 (48%)	114 (100%)	<0.001
	Frailty intervention (Oncogeriatrics)	4 (4%)	46 (41%)	<0.001
Tobacco dependency *n* (%)	Patients with tobacco dependency receiving specialist support / treatment	5/34 (15%)	30/33 (91%)	<0.001
Malnutrition	Screening for malnutrition	64/94 (68%)	114/114 (100%)	<0.001
	Intervention^a^ in moderate/high risk	1/9 (11%)	19/19 (100%)	<0.001
Alcohol dependency	Patients screened for high-risk alcohol intake	4/94 4%	114/114 100%	<0.001
	Appropriate alcohol intervention protocol followed	0/94 0%	114/114 100%	<0.001

a Nutritional intervention in moderate to high risk of malnutrition is written information on food fortification, provision of oral nutritional supplements and referral to specialist dietician.

A total of 230 hospital clinic appointments were required to reach a treatment decision in 94 pre-implementation patients vs 114 appointments in 114 patients in the post-implementation cohort (mean 2.4 ± 1.3 per patient vs one, *p* < 0.001, Table 4). The mean length of stay for surgical patients reduced from 9 days (±8) pre-implementation to 7 days (±7, *p* = 0.18) post-implementation.

**Table 4:** Outcomes and healthcare utilisation pre and post implementation of the GM One-stop Lung Cancer Clinic.

	Cohort	Pre-implementation of One Stop N = 94	Post-implementation of One Stop N = 114	*p*-value
Treatment modality	Surgical resection	69/94 (73%)	72/114 (63%)	0.116
	Curative-intent radiotherapy	25/94 (27%)	42/114 (37%)	0.116
Healthcare utilisation	Hospital appointments to reach treatment decision	230 in 94 patients	114 in 114 patients	
	Mean (±95% CI)	2.4 (±1.3)	1.0 (±0.0)	<0.001
	Median (IQR)	2 (1–3)	1 (1–1)	<0.001
Surgical length of stay (days)	Mean (±95% CI)	9 (±8)	7 (±7)	0.18
	Median (IQR)	7 (4–10)	6 (4–8)	0.18
Mortality	30-day mortality *n* (%)	0/94 0%	2/114 1.8%	0.50
	90-day mortality *n* (%)	4/94 (4.3%)	4/114 3.5%	>0.99
	12-month mortality *n*(%)	12/94 (12.8%)	4/114 3.5%	0.013

The mean ‘overall experience of care’ rating was 9.6/10. 90% (103/114) of patients considered it ‘very important’ to meet all specialists in one visit before deciding on treatment. Additionally, 88% (100/114) found the information provided in the clinic to be ‘very clear’, 93% (106/114) felt healthcare professionals listened to their concerns ‘very well’, 94% (107/114) felt ‘very supported’, and 80% (91/114) found making a treatment decision ‘very easy’ or ‘easy’.

In the pre-implementation cohort, 73% (69/94) of patients underwent surgical resection and 27% (25/94) underwent radiotherapy, vs 63% (72/114) and 37% (42/114) respectively in the post-implementation cohort. The 30-day, 90-day and 12-month mortality in the pre-implementation cohort was 0% (0/94) vs 1.8% (2/114, *p* = 0.50) post-implementation, 4.3% (4/94) vs 3.5% (4/114, *p* > 0.99) and 12.8% (12/94) vs 3.5% (4/114, *p* = 0.013, Table 4).

## Conclusion and next steps

The GM-OSLCC has significantly reduced the time from referral to treatment decision, with a median saving of 16 days and elimination of long waits. Furthermore, the 12-month mortality has significantly reduced (12.8% to 3.5%) and patients opting for surgery have a reduced length of stay. This may in part be due to the beneficial impact of shortened pathway times, but also due to substantial increases in prehabilitation uptake, frailty recognition and intervention, tobacco dependency treatment, malnutrition interventions and alcohol dependency interventions. Additional healthcare benefits include reduced hospital utilisation, with approximately 160 fewer appointments among 114 post-implementation patients. This has been delivered with an exceptional experience of care despite the inherently intense nature of multiple same-day consultations.

We conducted a PubMed search for ‘One Stop’ or ‘joint clinic’ models in cancer treatment across all tumour sites, but found no relevant publications. We did identify that one-stop models are more established for diagnostic pathways, such as in breast, prostate and upper gastrointestinal cancers, where diagnostic tests or ‘bundles’ are scheduled on the same day. However, these models do not typically involve the same setting for different treatment modalities where clinical equipoise exists.

The strengths of this analysis of the GM-OSLCC include well-defined objectives and measures between the two cohorts, which were clearly characterised. Limitations include the use of real-world data rather than RCT data or structured research analysis. However, an RCT would not have been feasible in this setting, and we believe that this case study provides substantial evidence of the impact of the service model. Another limitation is the potential lack of comparability between the pre-implementation and post-implementation cohorts, as they were from different timeframes, which could introduce some seasonal or temporal variation. Despite this, we believe that the overall impact on outcomes is minimal. Patients attending the GM-OSLCC had significantly worse functional status and physiological reserve than those assessed at the thoracic surgery HRMDT, which may indicate that more high-risk patients are now accessing comprehensive surgical assessments. This shift may also suggest that ‘less risky’ patients are being directed to routine surgical assessment, leaving the one-stop service for those with greater risk. These shifts could optimise surgical resection rates. Notably, the improvements observed in the post-implementation cohort are even more significant, considering their worse physiological and functional status compared with the pre-implementation cohort. It is important to note that the GM-OSLCC is a resource-intensive service. While we believe the patient benefits justify this investment, a formal health economic analysis is underway, and we aim to publish the return on investment results once completed.

The GM-OSLCC provides a case study of successful delivery of a complex cancer care pathway which is patient-centred and provided in a supportive environment. This could represent a transferable model of care for complex cancer pathways where there are opportunities for patient optimisation and challenging treatment decisions to be made.

## Data availability statement

All data generated or analysed during this study are included in this published article and its supplementary information files.

## Ethical approval and consent

This is an evaluation of a new service delivery model. Utilisation of the Health Research Authority’s decision tool indicates that this work is not research and does not require ethics committee approval. Written consent from participants was not required as it’s a service evaluation. All patient information was handled in compliance with GDPR and local governance protocols.

## Funding

This research did not receive any specific grant from funding agencies in the public, commercial, or not-for-profit sectors.

## CRediT authorship contribution statement

**Jonathan Hiu Nian Chung:** Writing – review & editing, Writing – original draft, Visualization, Methodology, Investigation, Formal analysis, Data curation, Conceptualization. **Kavita Sivabalah:** Writing – review & editing, Methodology, Data curation. **George Hulston:** Writing – review & editing, Data curation. **Patrick Goodley:** Writing – review & editing, Methodology, Formal analysis, Data curation. **Lisa Galligan-Dawson:** Writing – review & editing, Methodology, Conceptualization. **Sarah Lyon:** Data curation, Conceptualization. **Felice Granato:** Conceptualization. **Vijay Joshi:** Conceptualization. **Aleksander Mani:** Writing – review & editing, Conceptualization. **David Woolf:** Writing – review & editing, Conceptualization. **Kathryn Banfill:** Writing – review & editing, Methodology, Conceptualization. **Claire Barker:** Writing – review & editing, Conceptualization. **Jennifer King:** Writing – review & editing, Conceptualization. **Cassandra Ng:** Writing – review & editing, Conceptualization. **Helen Huddart:** Conceptualization. **Rebecca Stephens:** Data curation. **Siobhan Keegan:** Writing – review & editing, Methodology, Formal analysis, Data curation. **Karen Peplow:** Conceptualization. **Julie Watts:** Conceptualization. **Kirsty Rowlinson-Groves:** Data curation, Conceptualization. **Jack Murphy:** Conceptualization. **Kath Hewitt:** Formal analysis, Data curation, Conceptualization. **Matthew Evison:** Writing – review & editing, Writing – original draft, Methodology, Investigation, Formal analysis, Data curation, Conceptualization.

## Declaration of competing interest

The authors declare that they have no known competing financial interests or personal relationships that could have appeared to influence the work reported in this paper.

## Appendix Supplementary materials

Image, application 1
