# Supplementary material for: Fast-tracked and optimised: The impact of a one-stop lung cancer clinic on patient outcomes
Source: Future Healthc J. 2025 Sep 26;12(4):100474. doi: 10.1016/j.fhj.2025.100474 (PMC12590275; doi:10.1016/j.fhj.2025.100474)

Supplementary materials

Box 1. Absolute and relative indications for the GM One-stop Lung Cancer Clinic

Absolute indications:

If a patient fulfils these criteria then one-stop clinic appointment is mandated:

- BMI <= 20
- Proposed surgical option is pneumonectomy
- Performance status ≥2
- Clinical Frailty Score ≥5
- Established diagnosis of interstitial lung disease
- Post-operative predictive FEV1 <40%
- Post-operative predictive DLCO <40%
- Shuttle walk test <250m

Relative indications:

If a patient fulfils these criteria then one-stop clinic appointment could be considered

- Recent MI / CVA / PE (within the last 6 weeks)
- Thoracic revised cardiac risk index >=2 factors
- Previous irradiation to the thorax
- Chronic kidney disease stage 4-5 (eGFR <30)
- Severe PVD (claudication limiting functional capacity)
- Alcohol intake >20 units / week
- Previous head and neck surgery
- Any factor considered by the referring / receiving teams that makes an individual patient higher risk

Supplementary material 1

One-Stop
Lung Cancer Clinic

MFT Lung Cancer &
Thoracic Surgery Directorate

NEW PATIENT ASSESSMENT PROFORMA

NHS Manchester University NHS Foundation Trust

NHS The Christie NHS Foundation Trust

Greater Manchester Cancer

The Being You Centre @St Ann's

SECTION 3

Observations & Assessments

Weight: kg	BP: /	◯ Bloods taken
Height: cm	Pulse:	◯ ECG performed & reviewed
BMI:	Regular / irregular	◯ 30sec Sit to stand filmed<br>Result: ____
Haemoglobin (if known):	Respiratory rate:	◯ Get up & go filmed<br>Result: ____
Creatinine (if known):	Oxygen saturations: %	POC Pro-BNP<br>Result: ____
Albumin (if known):	Lying BP: / N/A	
	Standing BP: /	

Performance Status: (tick Box)

ECOG	Decription	Tick
0	Fully active, able to carry on all pre-disease performance without restriction	
1	Restricted in physically strenuous activity by ambulatory and able to carry out work of a light or sedentart nature e.g light house work, office work	
2	Ambulatory and capable of all selfcare but unable to carry out any work activities. Up and about more than 50% of waking hours	
3	Capable of only limited selfcare, confined to bed or chair more than 50% of waking hours	
4	Completely disabled. Cannot carry on selfcare, totally confined to bed or chair	

Nutrition screening:

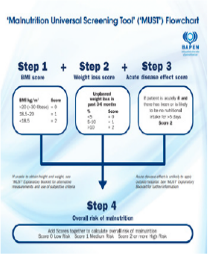

MUST score: ....

Confirm actions taken:
Medium risk:
◯ Provide 'Eating to be stronger' leaflet
◯ Consider provision of nutritional supplements
◯ Consider dietician referral (via GP/local hospital if required)

High risk
◯ Provide 'Eating to be stronger' leaflet
◯ Provide nutritional supplements
◯ Dietician referral (via GP/local hospital if required)

SECTION 4

Frailty & holistic care assessment

Clinical Frailty Score
To be performed in patients >=65 years old, tick the most appropriate score

Category	Clinical Frailty Scale	Tick
1	**Very Fit** - People who are robust, active, energetic and motivated. These people commonly exercise regularly. They are among the fittest fir their age.	
2	**Well** - People who are have no active disease symptoms but are less fit than category 1. Often, they exercise or are very active occasionally, e.g. seasonally.	
3	**Managing Well** - People whose medical problems are well controlled, but are not regularly active beyond routine walking.	
4	**Vulnerable** - While not dependant on others for daily help, often symptoms limit activities. A common complaint is being "slowed up", and/or being tired during the day.	
5	**Mildly Frail** - These people often have more evident slowing, and need help in high order IADLs (finances, transportation, heavy housework, medications). Typically, mild frailty progressively impairs shopping and walking outside alone, meal preparation and house work.	
6	**Moderately Frail** - People need help with all outside activities and with keeping house. Inside, they often have problems with stairs and need help with bathing and might need minimal assistance (cuing, standby) with dressing.	
7	**Severely Frail** - Completely dependant for personal care, from whatever cause (physical or cognitive). Even so, they seem stable and not at high risk of dying (within - 6 months).	
8	**Very Severely Frail** - Completely dependant, approaching the end of life. Typically, they could not recover even from a minor illness.	

Abbreviated Mental Test
(one point per correct answer):

Question	Tick if correct
Age	
Time (to nearest hour)	
Address for recall at the end of the test (**42 West Street**)	
Year	
Name of this place	
Correctly identify two people e.g. doctor/nurse	
Date of birth	
Year of First World War (1914-1918)	
Name of present Monarch	
Count backwards 20 to 1	

Indications for referral to the oncogeriatric service:

◯ Clinical Frailty Score ≥5
◯ Abbreviated Mental Test Score ≤6 (with no diagnosis of dementia)
◯ Any patient deemed at higher risk of post-operative delirium
◯ Geriatric syndromes – recurrent falls, new incontinence, inappropriate polypharmacy
◯ Patients under the age of 65 (CFS not validated) deemed to be frail by clinical team

SECTION 5

About the patient

Please tell us about yourself and the people who are important to you: *For example, who do you live with? Do you have a support network of friends and family to help you? Do you work? If so, is your employer able to be flexible when you're undergoing your treatment? Do you have young children, or other caring responsibilities? Discharge plans/transport.*

What are you most worried about right now? *Your treatment/surgery/radiotherapy, uncertainty, which could include worries about who will help with your children / people you care for whilst you are having your treatment, financial worries, worries about the treatment and side effects, how your treatment may affect your fertility.*

What are the most important things for us to take into consideration about you, right now, when planning your care? *(Your answer to this question will be included on the multi-disciplinary team meeting form if applicable, to inform decisions on planning your care.) For example, you may be keen to get treatment started as quickly as possible, or you may want more information about treatment or surgery options. You may have concerns about recovery time after surgery, and you may want us to know about any plans in the near future such as holidays, family weddings.*

SECTION 6

Medical History

Diagnosis & proposed operation

For lung cancer provide:

Staging: T: ☐ N: ☐ M: ☐ **Overall Stage:**

Location:

Right upper lobe ○ Right middle lobe ○ Right lower lobe ○ Left upper lobe ○ Left lower lobe ○

Presenting History:

Co-morbidities (tick box):

Respiratory	Tick	Cardiovascular disease	Tick	Neurological disease	Tick
COPD		Ischaemic heart disease		Dementia – Alzheimer's	
Emphysema		Congestive cardiac failure		Dementia – vascular	
Bronchiectasis		Aortic valve disease		Multiple sclerosis	
Interstitial Lung Disease		Mitral valve disease		Parkinson's disease	
Asthma		Atrial fibrillation/flutter		Epilepsy	
		Permanent pacemaker			
		Cerebrovascular disease			
		Peripheral vascular disease			
		Hypertension			
**Other**	**Tick**	**Mental Health Illness**	**Tick**	**Specific questions**	**Tick**
Diabetes – insulin dependent		Depression		Communication difficulties	
Diabetes – non-insulin dependent		Anxiety			
Chronic Kidney disease		Schizophrenia			
Hearing impairment		Bipolar disease			

Other co-morbidities & information on co-morbidities:

Anaesthetic history & assessment

Previous surgical History:

Any previous problems with anaesthesia:

Any previous post-operative complications or prolonged length of stay:

Any swallowing difficulties:
(if yes - please consider referal to SALT team)

○ SALT referral made

Smoking status:

○ Current smoker (smoked tobacco within the last 4 weeks)
○ Ex-smoker
○ Lifelong non-smoker (<100 cigarettes smoked in lifetime)

Current smokers are to be offered support by the CURE team. Provide brief advice as follows:

'Stop smoking is critically important prior to your treatment. Nicotine is the addictive substance in tobacco smoke but itself, is harmless. The danger and harms of smoking come from all the other chemicals in tobacco smoke. The best chance of stopping smoking is with the help of a specialist and either medications or providing nicotine in a safe (patches + gums/sprays) or significantly less harmful ways (vaping). Our stop smoking team will see you today and discuss all of these options.

○ Tick to confirm brief advice given
○ Tick to confirm CURE informed to review

Alcohol status:
Answer the questions below using a guide to estimate units

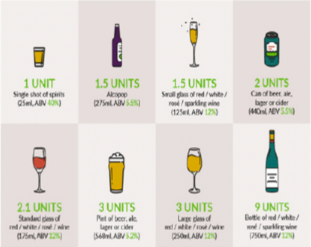

Questions	Scoring System					Score
	1	2	3	4	5	
How often do you have a drink containing alcohol?	Never	Monthly or less	2 - 4 times per month	2 - 3 times per week	4+ times per week	
How many units of alcohol do you drink on a typical day when you are drinking?	1 -2	3 - 4	5 - 6	7 - 9	10+	
How often have you had 6 or more units if female, or 8 or more if male, on a single occasion in the last year?	Never	Less than monthly	Monthly	Weekly	Daily or almost daily	

Scoring:

0-4 indicates lower-risk drinking – Feedback risk level to patient - no further action required

5-12 requires the following 7 questions to be completed to get a clearer picture

Questions	Scoring System					Score
	1	2	3	4	5	
How often during the last year have you found that you were not able to stop drinking once you had started?	Never	Less than monthly	Monthly	Weekly	Daily or almost daily	
How often during the last year have you failed to do what was normally expected from you because of your drinking?	Never	Less than monthly	Monthly	Weekly	Daily or almost daily	
How often during the last year have you needed an alcoholic drink in the morning to get yourself going after a heavy drinking session?	Never	Less than monthly	Monthly	Weekly	Daily or almost daily	
How often during the last year have you had a feeling of guilt or remorse after drinking?	Never	Less than monthly	Monthly	Weekly	Daily or almost daily	
How often during the last year have you been unable to remember what happened the night before because you had been drinking?	Never	Less than monthly	Monthly	Weekly	Daily or almost daily	
Have you or somebody else been injured as a result of your drinking?	No		Yes, but not in the last year		Yes, during the last year	
Has a relative or friend, doctor or other health worker been concerned about your drinking or suggested that you cut down?	No		Yes, but not in the last year		Yes, during the last year	
					Total	

Actions:

Overall score 8-15: Increasing risk: provide leaflet 'Advice About Alcohol and Your Health'. **Tick when action completed** ☐

Overall score 16-19: Higher risk: Refer to the Alcohol team on EPR selecting 'pre-operative' category. **Tick when action completed** ☐

Overall score ≥20: Dependence: Refer to the Alcohol team on EPR selecting 'pre-operative' category & discuss with alcohol team on 6572. Alcohol pathway to be commenced on admission. **Tick when action completed** ☐

Supplementary material 2

What you should bring on the day (checklist)

- A relative or friend
- All of your current medications (all tablets, medicines, injections and inhalers)
- Glasses, hearing aids, walking aids as required
- Personal contact numbers and email addresses
- A pen and paper to write information and questions down
- We will provide tea/coffee and biscuits but if you have any special dietary requirements, please bring drinks/snacks as required

A member of the nursing team will telephone you before the day to go through this information again and answer any questions you have about your upcoming visit to the One-Stop Lung Cancer Clinic.

If you require transport for your visit to our clinic, we will arrange this for you. If you have questions about this please call the Neil Cliffe Centre on 0161 291 4375.

Please use the space below to write any questions down you may have for the doctors and nurses in the clinic before or on the day:

We look forward to meeting you.
The One-Stop Lung Cancer Clinic Team

How to find the clinic

The clinic is located at:

The Neil Cliffe Centre, Entrance 12, Wythenshawe Hospital, Southmoor Road, Manchester, M23 9LT.

Tel: 0161 291 4375

Where should I park?

The Neil Cliffe centre is located at the back of Wythenshawe Hospital, near the Maternity Unit. There is a public car park, accessed via Floats Road, in front of the maternity department. From there, walk around the corner to the right of the maternity department and you will see the Neil Cliffe Centre entrance.

This is a photo of the Neil Cliffe centre where the clinic takes place:

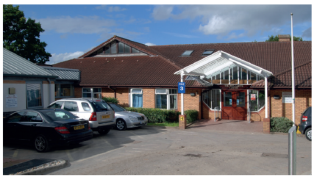

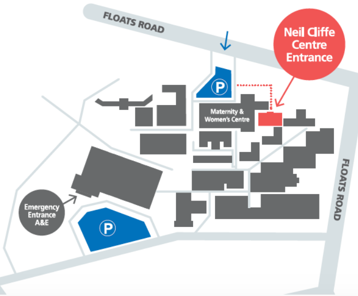

NHS

in Greater Manchester

One-Stop Lung Cancer Clinic

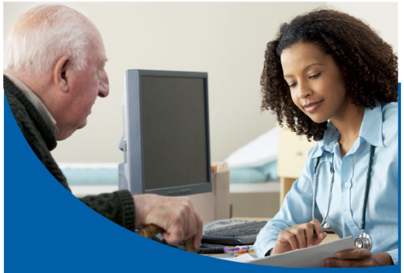

One-Stop Lung Cancer Clinic

PATIENT INFORMATION LEAFLET

The One-Stop Lung Cancer Clinic provides an opportunity for you and those closest to you, together with the doctors and nurses involved in your care, to consider what form of treatment will suit you best.

Decisions about your treatment

Often, a person is diagnosed with a lung cancer that could be treated in several different ways.

For example, lung cancer can sometimes be treated with:

- Surgery to remove the cancer, or;
- Radiotherapy which uses beams of radiation directed into the lungs to destroy the cancer

It is also often the case that one treatment is not significantly better than another treatment. So, it is important that your medical team helps you to understand the different treatments and the different advantages and disadvantages of each treatment.

There are also drug treatments, including chemotherapy, immunotherapy and targeted treatments that can be combined with your surgical or radiotherapy treatment plan. These treatments can be given before, alongside or afterwards and form part of your treatment plan.

It is very important to say, when lung cancer has been caught early then both surgery and radiotherapy are very effective treatments with very high success rates.

Purpose of the clinic

During your time with us in the clinic, we want to provide you with clear and easy to understand information about your treatment options so that we can decide together which treatment will suit you best.

It is not for us to tell you which treatment you can or can't have, but instead to support you to make the right decision for you. To help you with this, there may be a lot of information to discuss. You may also meet several different doctors and nurses.

The results of all the different tests you have already had, along with some further tests to be completed on the day of your clinic visit, will be reviewed by our team and discussed with you.

Our team

During the day you might meet some of the following members of our team:

Thoracic surgeon
A specialist that performs operations to remove cancers from the lung

Clinical oncologist
A specialist in treating lung cancer with radiotherapy

Medical oncologist
A specialist in treating lung cancer with drug treatments, also known as systemic anti-cancer treatments

Anaesthetist
A specialist in looking after patients before, during and after an operation

Lung cancer physician
A specialist in all aspects of lung cancer diagnosis and treatments

Thoracic or Macmillan nurse
Specialist nurses supporting patients with cancer

CURE team
A team of specialists helping patients that smoke to stop

Prehab4cancer team
Exercise practitioners that help patients prepare for cancer treatment

Oncogeriatrician
A specialist doctor that supports patients with cancer to improve all aspects of their health

The 'Being You' team from St Ann's Hospice
A team that provides a range of wellbeing tools available to support you

Please bring a family member or friend with you for support on the day, an extra pair of ears is always helpful!

What happens at the clinic

Clinic start time: 8am
Please arrive promptly for 8am as the first hour of your day will be busy. You can expect to be with us in the clinic for several hours (often the entire morning, sometimes longer if needed).

On your arrival, nursing staff will meet you and complete all the necessary outstanding tests and assessments. Please wear comfortable, loose clothing and your everyday shoes.

Next, your doctors and nurses will meet together to review and discuss the tests you have had done at your local hospital and when you first arrived at our clinic.

Meanwhile, you will have the opportunity to watch a range of information videos about lung cancer treatments and how to prepare for these treatments.

You will also meet any teams relevant to your needs, such as the specialist stop smoking team or a complimentary therapist from St Ann's Hospice.

Finally, you will have the chance to meet with the different doctors, discuss your treatment options and will be supported to make a decision about your treatment.

There is a lot of information to take in and you will be supported by our specialist nurses to ask any questions throughout the day.

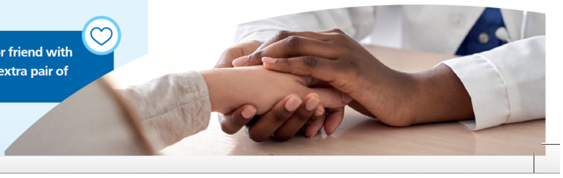

Supplementary material 3

Q12 Overall, please rate your experience of care from 1 - 10 (1 being the worst experience of care and 10 being the best experience of care)

Answered: 119 Skipped: 10

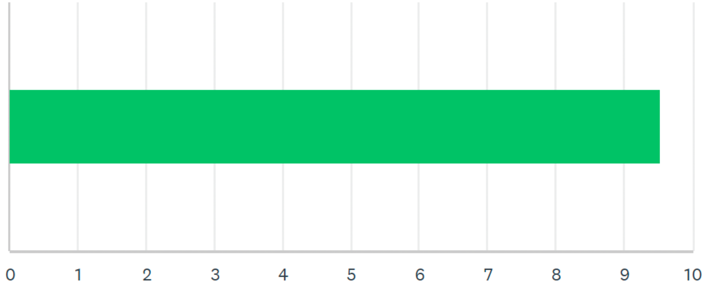

Supplement: Supplementary file 1 [file mmc1.pdf]
